# The Age Friendly Supermarket Index: a Delphi study and psychometric analysis

**DOI:** 10.3389/fpubh.2026.1891483

**Published:** 2026-08-28

**Authors:** Sok Leng Che, Wai In Lei, Sok Man Leong

**Affiliations:** 1 Nursing and Health Education Research Centre, Kiang Wu Nursing College of Macau, Macao, Macao SAR, China; 2 Nursing and Health Study Centre, Kiang Wu Nursing College of Macau, Macao, Macao SAR, China; 3 Research Management and Development Department, Kiang Wu Nursing College of Macau, Macao, Macao SAR, China

**Keywords:** age-friendly, Delphi, index, reliability, supermarket, validity

## Abstract

**Background and objectives:**

Person–environment interactions shape how individuals evaluate their surroundings, and perceived age friendliness influences intentions to age in place. Supermarkets, as essential components of daily urban life, play a critical role in supporting older adults’ community participation. This study aimed to develop and validate the Age Friendly Supermarket Index (AFSI), a comprehensive tool to assess and enhance supermarket age friendliness for older customers.

**Methods:**

Indicators were generated through a literature review, followed by two Delphi rounds, cognitive interviews, and pilot testing. The AFSI was validated using survey data from 387 older adults in Macao.

**Results:**

The finalized AFSI includes 33 indicators across three domains: (i) environmental design and customer service, (ii) convenience and safety assurance, and (iii) product arrangement and aisle planning. Exploratory and confirmatory factor analyses supported a three factor structure. The overall index showed excellent internal consistency (Cronbach’s *α* = 0.93), with strong reliability across domains (α = 0.90–0.92), explaining 54.96% of variance. Measurement invariance was confirmed across gender, age groups, and living status.

**Conclusion:**

The AFSI is a reliable and valid instrument for evaluating supermarket age friendliness and can guide improvements to support age-friendly shopping environments.

## 1 Introduction

The person–environment (P-E) interaction model emphasizes the dynamic process through which older adults adapt their personal capacities, including physical and psychological abilities, to the demands of the physical and social environment (1). Within this framework, the environment is considered a crucial factor in supporting older adults in maintaining their feelings of independence and autonomy throughout the ageing process (2). The World Health Organization’s Age-Friendly Cities framework operationalizes the P–E interaction into eight key characteristics of age-friendly cities (3), among which the domains of social participation and respect and social inclusion are particularly relevant to shopping activities among older adults. Social participation encompasses the provision of age-friendly facilities and services, the prevention of social isolation, and the promotion of intergenerational integration within the community. The domain of respect and social inclusion emphasizes designs and services that respond to the needs of older adults, enabling them to engage meaningfully in both community life and economic activities. One perceived the friendliness affects their intention to age in place (4). All of this matches this aim of supporting older people to age in place while continuing their daily life and performing their roles.

Shopping is an essential component of daily life living in community. In urban settings, older adults have increasingly considered supermarkets as one of their main sources of food and daily necessities, attracted by improved shopping environments, perceived food quality, and greater product variety (5, 6). Shopping is closely associated with older adults’ nutritional intake, self-care capacity, and the construction of positive social identities, and it has also been shown to predict survival among older populations (7–9). Moreover, shopping is an indicator of independence, and has been utilized as a form of rehabilitation, as it can contribute to the maintenance and improvement of physical and cognitive functions (10, 11). Therefore, accessing supermarkets involves multiple domains of the WHO’s AFC framework, ranging from outdoor spaces and buildings, such as accessible building design and the availability of public toilets, to social participation, including the accessibility of activities, facilities, and settings, the reduction of social isolation, and the potential facilitation of community integration.

Research has shown that older shoppers frequently encounter difficulties when shopping in supermarkets, including difficulty locating desired products, shelves that are too high to reach, unclear signage, and malfunctioning or poorly maintained equipment (12, 13). Moreover, older adults’ shopping behaviors are shaped not only by in-store experiences but by the entire shopping journey, from pre-trip preparation and transportation to the store, to in-store navigation and the return home (5, 14). These interconnected stages of shopping collectively influence older adults’ store choice and shopping patterns.

To date, no specific index exists for evaluating the age-friendliness of supermarkets. Although general commercial guidelines, such as Age UK’s “Top Tips” for age-friendly businesses (15), address issues including safe shopping environments, customer service, and digital design, they remain overly generic and lack operational specificity, limiting their immediate applicability in practice. Similarly, Hong Kong’s Criteria for an “Age-friendly Shopping Mall” focus on spatial planning and service provision but are tailored to shopping malls (16). Given the substantial differences in store scale, layout, shopping patterns of customers, and operational demands between shopping malls and supermarkets, as well as the lack of operational detail and measurable criteria, substantially limits their usefulness for systematic evaluation or practical implementation in supermarket settings. Another study conducted in Taiwan focused on the planning and design of age-friendly supermarkets. Taking into account age-related changes in physical and psychological characteristics, consumer behaviors, and the demands of age-friendly environments, the study synthesized a set of spatial design principles for older consumers. These principles include safety, inclusiveness, legibility, accessibility, and health, and were operationalized across nine aspects of supermarket planning and design, comprising a total of 33 detailed items (17). However, these principles emphasize the physical environment only. While improvements in physical and spatial design can alleviate difficulties arising from age-related physical decline, the consideration of interactions between staff and older customers, as inclusive and positive social interactions play an equally crucial role in enhancing older adults’ well-being (12, 18). Thus, whereas physical environments primarily address functional limitations, socially supportive services contribute to improved mental health and overall shopping experiences among older adults.

While these frameworks are valuable, they function primarily as general guidelines and place a strong emphasis on the physical environment. As a result, they fail to capture both the tangible and the intangible dimensions of the overall shopping experience. Moreover, these guidelines have not undergone empirical validation, making it difficult to assess their effectiveness in determining whether a supermarket is age-friendly. Their applicability across different cultural contexts is also uncertain, underscoring the need for locally grounded instruments. In Macao, the supermarket landscape presents unique contextual characteristics that further underscore the need for a locally grounded assessment tool. Supermarkets are commonly embedded within dense residential neighborhoods and operate within highly constrained floor areas due to limited urban space and high population density. These spatial limitations shape store layout, aisle width, product display capacity, and the feasibility of implementing age-friendly modifications. As a result, mall-based age-friendly guidelines are difficult to apply directly to Macao’s supermarket settings, reinforcing the need for an instrument tailored to the city’s compact retail environment. Therefore, this study aimed to develop and validate an Age-Friendly Supermarket Index (AFSI) to provide a standardized and empirically tested tool for evaluating the age-friendliness of supermarkets in future research and practice.

## 2 Methods

The development of Age-Friendly Supermarket Index (AFSI) involved five stages: (1) item formulation to identify potential items from literature; (2) two rounds of Delphi survey for consensus on items, terminology, and domains; (3) cognitive interviews with residents aged ≥ 60 in Macao; (4) pilot testing; (5) formal implementation. This study adopted the definition commonly used in Hong Kong and mainland China, in which a supermarket is defined as a self-service retail establishment that sells fresh foods, non-fresh food items, beverages, and household goods (19, 20).

### 2.1 Items formulation/ identification of the initial set of relevant AFSI

**Figure 1:**
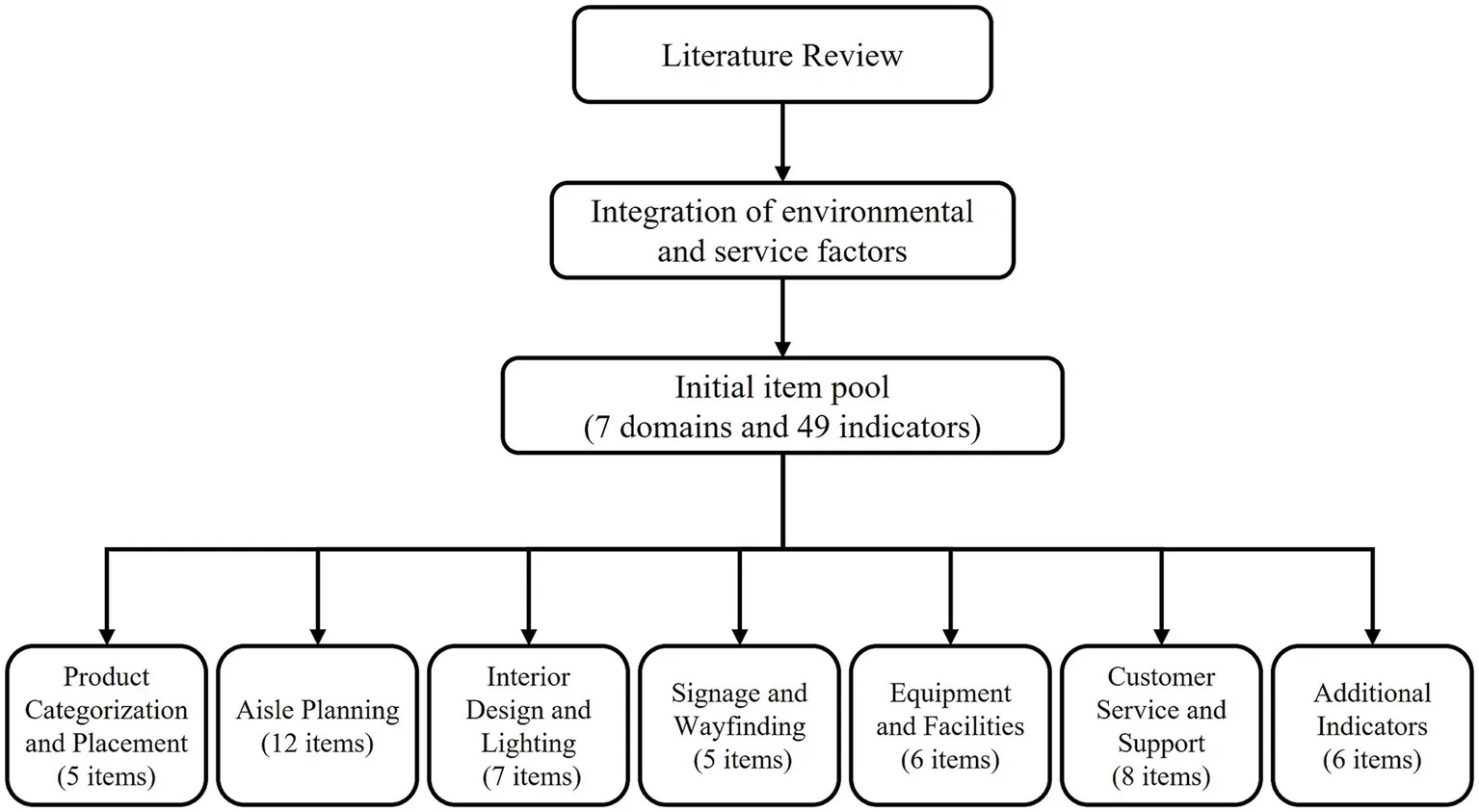
Conceptual framework for initial AFSI item development.

Through literature review, it was found that Lung (17) focused solely on the shopping environment for older adults, while Yin et al. (13) addressed facilities and services. However, the overall shopping experience is the result of the interaction between the environment and services and must consider all aspects of their impact on older adults. Therefore, the initial item pool was primarily developed by synthesizing the issues raised by Yin et al. (13) and Lung (17) regarding age-friendly supermarkets, resulting in 7 domains and a total of 49 indicators (see Figure 1).

### 2.2 Delphi survey

A diverse panel of 16 experts was convened to participate in two rounds of the Delphi process, consistent with recommendations found in the literature (21, 22). The expert panel included experts in the field of geriatric nursing education, geriatric social workers, an occupational therapist, a medical doctor affiliated with a community service organization, an interior designer specialized in age-friendly home design, members of the Commission for Older Citizen’s Affairs, and members of the Community Service Advisory Councils, consultative bodies established by the Macao Special Administrative Region Government to strengthen direct communication between the government and the community. A detailed description of the study’s purpose and evaluation instructions were provided.

A Delphi questionnaire was constructed and disseminated to the expert panel in order to collect their informed perspectives. The experts were asked to evaluate the importance of each domain and indicator separately, using the following scale: 1 = Not at all important, 2 = Slightly important, 3 = Moderately important, 4 = Important, 5 = Very important. The experts were instructed to select the appropriate box for each item. The Delphi method is an iterative, multistage process designed to synthesize individual opinions into group consensus. In addition to rating each item, qualitative comments from the experts were also collected and, in the second round, aggregated feedback was provided to all participants. An open-ended “Revisions and Suggestions” column was provided after each indicator to allow for comments regarding inaccurate or inappropriate wording (23). Additionally, an “Additional Recommendations” section was included after each domain to give suggestions for the addition of indicators.

The reliability of the Delphi method is evaluated through three indicators: experts’ positive coefficient, expert authority coefficient, and coordination coefficient. Among these, the experts’ positive coefficient serves as a measure of the effective response rate to the consultation questionnaire, thereby indicating the credibility and scientific rigor of the results. The expert authority coefficient (Cr) is determined by two factors: the judgment coefficient (Ca) and the familiarity coefficient (Cs) (24). The score of Ca represents the extent to which various bases for evaluation influence experts’ assessments of the indicators. The four bases for judgment include practical experience, theoretical analysis, references, and intuition; the degree of influence is assigned values as shown in Table 1. On the other hand, Cs is determined by the experts’ self-assessed familiarity with the topic, rated on a five-point Likert scale ranging from very familiar to very unfamiliar, with corresponding values of 1, 0.75, 0.5, 0.25, and 0, respectively. Cr is calculated as Cr = (Ca + Cs)/2, a Cr value >0.7 is considered to indicate acceptable reliability (25). The coordination coefficient, assessed using Kendall’s W concordance coefficient test, measures the degree of agreement among experts regarding the importance of each indicator. This coefficient evaluates the consistency of scores given by n experts across K indicators, with values ranging from 0 to 1. A statistically significant Kendall’s W test result indicates that consensus has been achieved among the experts.

**Table 1:** Value assignment for judgment coefficient (Ca).

Bases for judgment	Degree of influence		
	High	Medium	Low
Practical experience	0.5	0.4	0.3
Theoretical analysis	0.3	0.2	0.1
References	0.1	0.1	0.1
Intuition	0.1	0.1	0.1

In this study, the methods for calculating each indicator score also included: (1) determining the mean (M) and standard deviation (S) of scores for each indicator; (2) calculating the consensus rate, defined as the proportion of indicator scores of 4 or 5 (K); and (3) computing the coefficient of variation (CV=S/M), which reflects the variability in experts’ scoring for each indicator. A lower CV indicates greater consensus among experts. Competencies were accepted if, for all three criteria (importance, feasibility, and sensitivity), the mean scores (M) were at least 4.0, the consensus rate (K) reached 70%, and the CV was 0.25 or less (26).

### 2.3 Cognitive interview

After integrating expert feedback and implementing subsequent revisions, cognitive interviews were conducted to identify any phrases or terminology that might introduce ambiguity. These interviews took place in September 2025. Participants were purposively selected to ensure diversity across gender, age, and educational background. The participants consisted of 20 Macao residents aged 60 years or older.

### 2.4 Pilot test

A total of 20 participants were recruited for the pilot test to assess item clarity, response format, and feasibility of administering the preliminary 41-item scale, yielding Cronbach’s alpha of 0.881. The pilot was designed primarily as a qualitative and procedural check rather than for full psychometric evaluation or item-level analysis.

### 2.5 Formal implementation

A cross-sectional survey was conducted to recruit Macao residents aged 60 years or older. Data was collected using a structured questionnaire that included the AFSI and items pertaining to participants’ socio-demographic characteristics, such as age, gender, educational attainment, marital status, living arrangement, and health status. The AFSI consisted of 41 items (39 items for supermarkets with single floor, and 2 items pertaining to stairways and escalators), and respondents were instructed to rate each item on a five-point Likert scale ranging from 1 (strongly disagree) to 5 (strongly agree).

Eligibility criteria for participation required individuals to be current residents of Macau, aged 60 years or above, capable of providing informed consent, and comprehending Cantonese or Mandarin. Recruitment and data collection were carried out from October to December 2025.

Convenience and snowball sampling methods were utilized, leveraging online advertisements, social media platforms, and activity centers for older adults. Study posters containing a brief description and a link to the questionnaire were distributed to local social service organizations and colleges through platforms including Facebook, WhatsApp, and WeChat. Interested participants accessed the questionnaire via the link provided, where they could indicate informed consent prior to completing the survey. For those unable to navigate the online survey, trained interviewers administered face-to-face interviews to facilitate data collection.

### 2.6 Statistical analysis and index evaluation

Data coding was performed in Microsoft 365 Excel. Demographic variables were categorized and reported as frequencies and percentages. Data management, distribution analysis, and exploratory factor analysis (EFA) were carried out using the Statistical Package for the Social Sciences (SPSS, version 30), while confirmatory factor analysis (CFA) was conducted with Amos (version 29.0). To assess interpretability, distribution analysis included calculations of the median, range, and interquartile range were performed.

EFA was performed to examine the scale’s dimensionality and internal consistency. Factors with eigenvalues greater than one were extracted using principal component analysis (PCA), applying direct oblimin rotation to account for assumed correlations among factors (27). PCA was selected because the primary aim of the exploratory phase was to reduce the initial item pool and identify coherent item groupings, rather than to estimate latent constructs. PCA is widely used in early-stage instrument development to identify preliminary dimensional structures before confirmatory factor analysis is conducted (28). The adequacy of the dataset for factor analysis was determined by the Kaiser-Meyer-Olkin (KMO) measure and Bartlett’s test of sphericity; a KMO value above 0.70 and statistically significant indicated suitability for PCA (27). Satisfactory rotated factor loadings were defined as those greater than 0.4 on the primary factor (29). Parallel analysis (PA) was conducted to compare eigenvalues. Internal consistency reliability was evaluated using Cronbach’s alpha and McDonald’s omega. Composite reliability (CR), average variance extracted (AVE), and inter factor correlation were evaluated to establish convergent and discriminant validity. Models were considered acceptable when CR exceeded 0.70 and AVE was greater than 0.50 (30).

Confirmatory factor analysis (CFA) was conducted to evaluate the model’s goodness of fit using multiple fit indices, including the comparative fit index (CFI), goodness-of-fit index (GFI), Tucker–Lewis index (TLI), root mean square error of approximation (RMSEA), and standardized root mean square residual (SRMR). An acceptable model fit was indicated by CFI, GFI, and TLI values greater than 0.90, RMSEA values below 0.08, and SRMR values below 0.09 (31). All values were estimated using the maximum likelihood method (32). Given the early stage of instrument development and the need to retain adequate statistical power, EFA and CFA were conducted on the same dataset. This approach is commonly used in preliminary scale development when separate samples are not feasible.

After establishing an acceptable measurement model, multigroup confirmatory factor analysis (MGCFA) was performed to examine measurement invariance across subgroups (33). Configural, metric, and scalar invariance were sequentially tested. Measurement invariance was considered supported when changes in CFI (ΔCFI), TLI (ΔTLI), RMSEA (ΔRMSEA), and SRMR (ΔSRMR) were all less than 0.01 (34). Statistical significance was set at *p* < 0.05 for all analyses.

## 3 Results

### 3.1 Delphi survey

Round 1 of the Delphi survey was conducted in June 2025, followed by Round 2 in July 2025. The response rates for both rounds were 100%. The demographic and professional characteristics of the experts are presented in Table 2. A total of 16 experts from diverse professional backgrounds participated in the study. All experts were based in Macao and Hong Kong, and the majority had more than 10 years of professional experience (range: 5–60 years; mean ± SD = 19.44 ± 12.71 years). The expert authority coefficient (Cr) was 0.87, exceeding the accepted threshold of 0.70, indicating that the results of the expert consultation were reliable.

**Table 2:** Characteristics of participants in Delphi survey (n = 16).

Characteristics	*n*	%
Gender		
Male	9	56.3%
Female	7	43.8%
Education level		
Bachelor	2	12.5%
Master	12	75.0%
Doctor	2	12.5%
Years of experience		
5–10	5	31.3%
11–20	7	43.8%
> = 21	4	25.0%
Professional discipline		
Social work	5	31.3%
Nurse	3	18.8%
Medicine	1	6.3%
Occupational therapy	1	6.3%
Public health	1	6.3%
Public administration	3	18.8%
Interior design	1	6.3%
Community organizer	1	6.3%

A total of 7 domains and 49 indicators were extracted from literature to form an initial list. In round 1, 8 indicators did not achieve the predefined level of consensus (*K* < 70%). A total of 24 new items were proposed, and 61 comments were received. The suggestions primarily focused on revising negatively worded items to positive statements, refining wording, reallocating items to more appropriate domains, and addressing overlapping content. Following qualitative analysis, 1 domain and 9 items were removed, 2 domains and 23 items were reworded, 7 items were reorganized and merged; and 6 new items were added. Consequently, 6 domains comprising 45 items were retained for round 2. In Round 2, all 45 items met the 70% agreement criterion. A total of 55 comments were analyzed, resulting in the rewording of 16 items and the deletion of 4 items. This process yielded a final set of six domains comprising 41 items for the cognitive interview stage (Table 3).

**Table 3:** The initial list and the results of round 1 and 2 in the AFSI.

Survey version indicators	Initial list number	Round 1	Round 2
Product categories are standardized with relatively fixed placement locations	A1	^	^
Frequently purchased items for older adults are prominently displayed	A2	^	^
Frequently purchased items for older adults are placed at easily accessible heights	A3	^	^
Product variety meets daily living needs	A4	^	#
A dedicated section for older adult essentials (e.g., anti-slip mats, non-slip slippers, prescription pill organizers, adult diapers) is available to meet shopping needs	A5	^	#
A dedicated section for healthy foods (e.g., nutritional milk powder, low-sugar, low-salt, low-fat, high-fiber foods) is available with diverse options provided		+	#
Entrances, exits, and in-store aisles are flat or barrier-free (e.g., gentle ramps)	B1	#	^
The narrowest point of entrances/exits is wide enough for wheelchair access		+	^
*The floor in the store is easy to slip on	B2	@	
In-store floors are dry or equipped with anti-slip surfaces	B3	^	#
*The floor in the fresh food area is slippery	B4	@	
In-store aisles are wide enough for wheelchairs or shopping carts, with no merchandise blocking pathways	B5	^	^
*The checkout lane is not too narrow or too wide	B6	^	@
Checkout areas provide sufficient waiting space	B7	^	#
All shelves within reach for older adults to access and retrieve items	B8	^	^
Stairs leading to the supermarket entrance or inside the store are safe (e.g., appropriate height, width, and depth; equipped with anti-slip strips or warning tape and sturdy handrails)	B9	^	#
*The stairs inside the store are safe	B10	@	
*The store stairs have safe, strong handrails	B11	@	
The escalators leading to the supermarket entrance or inside the store are safe (e.g., equipped with emergency stop buttons, warning tape at activation points, and operating at a slower speed). (Select “Not applicable” if no escalators are present.)	B12	^	#
Shelves and walls are non-reflective	C1	^	#
*The store has little echo and no noisy sounds	C2	#	@
Clear warnings are posted in wet goods areas (where seafood, meat, or vegetables are sold) or on slippery surfaces	C3	^	^
Store temperature is at a comfortable level	C4	#	#
Indoor lighting is sufficient and evenly distributed	C5	#	#
*Different product sections in the store are identified by colors.	C7	#	@
Air circulation is adequate and there are no unpleasant odors	E3	#	#
In-store signage is clear and easy to understand	D1	^	#
Signage font size is sufficiently large and posted at a height suitable for older adults’ vision	D3	^	#
Aisle markings and directional guidance are clear, directing customers to specific areas (e.g., checkout counters, entrances/exits)	D2	^	^
Product prices and names are clearly and legibly displayed, with signage matching the actual location of displayed items	D4	#	#
Emergency exit locations are clearly marked with sufficiently bright indicator lights	C6, D5	^	#
Key areas (e.g., restrooms, service counters, entrances/exits) feature clear signage supplemented with pictograms		+	#
Adequate supply of shopping baskets or carts	E1	^	^
Lightweight shopping baskets or effort-saving shopping carts	E2	^	#
*Magnifying glasses are provided in the product display area	E4	^	@
*Self-service lockers are available in sufficient number	E5	@	
Freezer sections are not too deep, making it easy to retrieve items	E6	^	^
The store provides an adequate number of rest chairs placed in convenient locations, safe and easy for seniors to use (e.g., equipped with armrests, appropriately sized)	G3	^	^
Emergency call buttons or staff summoning devices are clearly marked and easily accessible	G4	^	#
Accessible restroom facilities adequately meet user needs	G1	^	^
First aid kits and automated external defibrillators (AEDs) are placed in conspicuous locations	G5	^	^
Promotions and discounts are easy to find	F1	^	#
Promotional content is clear and accurate		+	#
*The store has an online shopping platform	F2	@	
Reasonable wait times at checkout	F4	^	#
Both self-checkout and staffed registers are available, with diverse payment options (cash, e-payments, credit cards, etc.)	F6	^	#
*The plastic bags are difficult to open	F5	@	
Delivery services meet customer needs	F3	#	#
Generous discounts for older adults	F7	^	#
Staff are friendly and proactively offer assistance (e.g., locating products, helping with self-checkout machines)	F8	^	^
Priority checkout lanes meet older adults’ needs		+	#
In-store audio announcements are clear and at an appropriate volume		+	^
*A baby changing room is provided	G2	@	
*Temporary child-care service is provided	G6	@	

# no change; @ removed; ^ combined or revised; + added. A. Product Classification and Display; B. Passageway Planning; C. interior Design and Lighting; D. Signage and Guidance; E. Equipment and Facilities; F. Software Services; G. Additional Indicator. * Not included in survey version.

### 3.2 Cognitive interview

The primary revisions made during the cognitive interview phase focused on improving the fluency and clarity of sentence structures. For example, the term “結帳” was revised to “付款” to enhance conversational naturalness. In addition, brief explanations were added for terms less commonly used in daily life; for instance, “濕貨區” was clarified as “an area selling seafood, meat, or vegetables.” As no further modifications were deemed necessary, the instrument was finalized and subsequently adopted for the pilot testing phase.

### 3.3 Participant characteristics

A total of 387 valid responses were collected. The majority of participants were female (82.2%), aged between 60 and 69 years (47.8%), had an education level of high school or below (83.5%), were married or cohabiting (58.7%), did not live alone (67.7%), and were retired (76.2%). In addition, most respondents reported fair self-rated health (58.9%), had at least one chronic disease (87.9%), and were able to walk independently (92.5%). Only a very small proportion of participants reported instrumental activities of daily living (IADL) disability (Table 4).

**Table 4:** Socio-demographic characteristics (n = 387).

Participant characteristics	*n*	%
Gender		
Male	69	17.8
Female	318	82.2
Age (years)		
60–69	185	47.8
70–79	150	38.8
> = 80	52	13.4
Education level		
Primary school or below	139	35.9
Secondary school	184	47.5
Bachelor’s degree or above	64	16.5
Marital status		
Not married or cohabiting	160	41.3
Married or cohabiting	227	58.7
Religion		
No	165	42.6
Yes (religious or folk beliefs)	222	57.4
Living status		
Alone	125	32.3
Living with others	262	67.7
Monthly income (MOP)		
0–5,000	180	46.5
5,001–10,000	112	28.9
> = 10,001	95	24.5
Self-rated health		
Fair/ bad/ very bad	273	70.5
Good/ very good	114	29.5
Chronic disease		
None	47	12.1
Yes	340	87.9
Walking aide		
No	358	92.5
Yes	29	7.5
IADL disability		
Shopping	7	1.8
Managing transportation	4	1.0
Meal preparation	2	0.5
Housekeeping	5	1.3
Laundry	12	3.1
Telephone use	1	0.3
Handling medications	10	2.6
Handling finances	1	0.3

### 3.4 Item and distribution analysis

Item analysis (Table 5) indicates all items possess strong discriminatory power between extreme groups and can effectively differentiate between high- and low-scoring participants. Furthermore, the correlation between all items and the overall scale was significant (Table 6), suggesting that all items measure the same underlying construct as the others. Lastly, the value of Cronbach’s *α* if item deleted did not change the overall Cronbach’s α, implying that removing any item would not improve the internal consistency of the scale, and would not change the reliability of the overall scale. However, the correlation matrix also revealed items 29 to 32, 35, 38, and 39 did not correlate with most items. The strongest correlation in the matrix is observed between item 8 and item 9 (*r* = 0.833, *p* < 0.001).

**Table 5:** Item analysis (*n* = 387).

Item	Item discrimination	Corrected item-total correlation coefficients	Cronbach’s α if item deleted
1. Product categories are standardized with relatively fixed placement locations	−7.35***	0.54	0.96
2. Frequently purchased items for older adults are prominently displayed	−11.64***	0.63	0.96
3. Frequently purchased items for older adults are placed at easily accessible heights	−13.37***	0.66	0.96
4. Product variety meets daily living needs	−9.67***	0.49	0.96
5. A dedicated section for older adult essentials (e.g., anti-slip mats, non-slip slippers, prescription pill organizers, adult diapers) is available to meet shopping needs	−12.15***	0.57	0.96
6. A dedicated section for healthy foods (e.g., nutritional milk powder, low-sugar, low-salt, low-fat, high-fiber foods) is available with diverse options provided	−12.13***	0.61	0.96
7. Entrances, exits, and in-store aisles are flat or barrier-free (e.g., gentle ramps)	−14.82***	0.66	0.96
8. The narrowest point of entrances/exits is wide enough for wheelchair access	−13.65***	0.61	0.96
9. In-store floors are dry or equipped with anti-slip surfaces	−15.43***	0.72	0.96
10. In-store aisles are wide enough for wheelchairs or shopping carts, with no merchandise blocking pathways	−18.03***	0.71	0.96
11. Checkout areas provide sufficient waiting space	−16.27***	0.68	0.96
12. All shelves within reach for older adults to access and retrieve items	−17.33***	0.70	0.96
13. Stairs leading to the supermarket entrance or inside the store are safe (e.g., appropriate height, width, and depth; equipped with anti-slip strips or warning tape and sturdy handrails) (n = 162)	−12.68***	0.73	0.96
14. The escalators leading to the supermarket entrance or inside the store are safe (e.g., equipped with emergency stop buttons, warning tape at activation points, and operating at a slower speed) (n = 162)	−11.96***	0.70	0.96
15. Shelves and walls are non-reflective	−11.65***	0.58	0.96
16. Clear warnings are posted in wet goods areas (where seafood, meat, or vegetables are sold) or on slippery surfaces	−14.11***	0.58	0.96
17. Store temperature is at a comfortable level	−11.90***	0.59	0.96
18. Indoor lighting is sufficient and evenly distributed	−13.20***	0.66	0.96
19. Air circulation is adequate and there are no unpleasant odors	−13.67***	0.68	0.96
20. In-store signage is clear and easy to understand	−15.14***	0.73	0.96
21. Signage font size is sufficiently large and posted at a height suitable for older adults’ vision	−17.38***	0.72	0.96
22. Aisle markings and directional guidance are clear, directing customers to specific areas (e.g., checkout counters, entrances/exits)	−15.16***	0.70	0.96
23. Key areas (e.g., restrooms, service counters, entrances/exits) feature clear signage supplemented with pictograms	−14.58***	0.63	0.96
24. Product prices and names are clearly and legibly displayed, with signage matching the actual location of displayed items	−14.38***	0.66	0.96
25. Emergency exit locations are clearly marked with sufficiently bright indicator lights	−13.65***	0.67	0.96
26. Adequate supply of shopping baskets or carts	−11.13***	0.56	0.96
27. Lightweight shopping baskets or effort-saving shopping carts	−12.32***	0.61	0.96
28. Freezer sections are not too deep, making it easy to retrieve items	−10.04***	0.62	0.96
29. The store provides an adequate number of rest chairs placed in convenient locations, safe and easy for seniors to use (e.g., equipped with armrests, appropriately sized)	−7.31***	0.40	0.96
30. Emergency call buttons or staff summoning devices are clearly marked and easily accessible	−7.41***	0.38	0.96
31. Accessible restroom facilities adequately meet user needs	−6.95***	0.45	0.96
32. First aid kits and automated external defibrillators (AEDs) are placed in conspicuous locations	−7.21***	0.38	0.96
33. Promotions and discounts are easy to find	−12.42***	0.57	0.96
34. Promotional content is clear and accurate	−12.61***	0.62	0.96
35. Generous discounts for older adults	−13.42***	0.61	0.96
36. Reasonable wait times at checkout	−12.98***	0.62	0.96
37. Both self-checkout and staffed registers are available, with diverse payment options (cash, e-payments, credit cards, etc.)	−11.78***	0.58	0.96
38. Priority checkout lanes meet older adults’ needs	−11.15***	0.58	0.96
39. Delivery services meet customer needs	−9.57***	0.53	0.96
40. In-store audio announcements are clear and at an appropriate volume	−12.50***	0.52	0.96
41. Staff are friendly and proactively offer assistance (e.g., locating products, helping with self-checkout machines)	−11.36***	0.54	0.96
Total Scale			0.96

***p < 0.001.

**Table 6:** Correlation between items and AFSI mean (*n* = 387) (*r*).

Item	Mean	Item	Mean	Item	Mean	Item	Mean
1	0.446***	11	0.698***	21	0.718***	31	0.389***
2	0.608***	12	0.649***	22	0.689***	32	0.397***
3	0.613***	13	0.753***	23	0.625***	33	0.562***
4	0.485***	14	0.719***	24	0.691***	34	0.598***
5	0.565***	15	0.564***	25	0.624***	35	0.578***
6	0.572***	16	0.576***	26	0.586***	36	0.632***
7	0.625***	17	0.576***	27	0.656***	37	0.575***
8	0.622***	18	0.642***	28	0.581***	38	0.551***
9	0.654***	19	0.673***	29	0.385***	39	0.477***
10	0.700***	20	0.732***	30	0.400***	40	0.569***
						41	0.593***

*** *p* < 0.001.

### 3.5 Exploratory factor analysis

The KMO measure was 0.944, and Bartlett’s test of sphericity was significant ( $\chi_{528}^{2}$ =7769.92, *p* < 0.001), indicating that the correlation matrix was appropriate for factor extraction. Principal component analysis (PCA) initially identified three factors. However, parallel analysis (PA) indicated a three-factor solution (Table 7). Accordingly, principal component analysis (PCA) with direct oblimin rotation was constrained to a three-factor solution, as the presence of correlations among items was confirmed by the correlation analysis (Table 6). The items collectively explained 54.96% of the total variance. The overall Cronbach’s alpha coefficient was 0.93, with subscale values of 0.92 for factor 1, 0.90 for factor 2, and 0.90 for factor 3. Similar results were observed for the Omega coefficients, indicating satisfactory internal consistency for both the overall scale and its subscales. The AVEs of the three factors were 0.39, 0.59, and 0.45, and CRs were 0.91, 0.91, and 0.91, respectively (Table 8). The correlation between factors were all <0.85, suggesting good discriminant validity (Table 9).

**Table 7:** Result of parallel analysis.

Root	Raw data	Means	Prcntyle
1	12.330286	1.792035	1.916819
2	2.634320	1.678377	1.759379
3	1.914664	1.593684	1.664392
4	1.470960	1.527531	1.586203
5	1.052290	1.456871	1.506336
6	1.008595	1.392207	1.440068
7	0.849488	1.337879	1.382765
8	0.762711	1.279290	1.323992
9	0.745176	1.237139	1.269036
10	0.716359	1.191424	1.232360

**Table 8:** Exploratory factor analysis and convergent validity of the AFSI.

Items	Factor Loadings			Communalities
	F1	F2	F3	
20. In-store signage is clear and easy to understand.	0.76	−0.07	0.12	0.68
28. Freezer sections are not too deep, making it easy to retrieve items	0.74	−0.18	0.00	0.52
18. Indoor lighting is sufficient and evenly distributed	0.72	−0.28	0.18	0.69
22. Aisle markings and directional guidance are clear, directing customers to specific areas (e.g., checkout counters, entrances/exits)	0.69	0.06	0.05	0.55
17. Store temperature is at a comfortable level	0.69	−0.16	0.03	0.48
24. Product prices and names are clearly and legibly displayed, with signage matching the actual location of displayed items	0.67	−0.05	0.17	0.61
19. Air circulation is adequate and there are no unpleasant odors	0.66	−0.15	0.21	0.63
21. Signage font size is sufficiently large and posted at a height suitable for older adults’ vision	0.65	0.07	0.14	0.58
15. Shelves and walls are non-reflective	0.65	−0.28	0.17	0.57
16. Clear warnings are posted in wet goods areas (where seafood, meat, or vegetables are sold) or on slippery surfaces	0.59	0.21	−0.09	0.39
23. Key areas (e.g., restrooms, service counters, entrances/exits) feature clear signage supplemented with pictograms.	0.57	0.27	−0.02	0.45
25. Emergency exit locations are clearly marked with sufficiently bright indicator lights	0.55	0.24	0.03	0.44
36. Reasonable wait times at checkout	0.54	0.18	0.10	0.45
40. In-store audio announcements are clear and at an appropriate volume	0.51	0.18	0.04	0.36
41. Staff are friendly and proactively offer assistance (e.g., locating products, helping with self-checkout machines)	0.41	0.10	0.24	0.38
37. Both self-checkout and staffed registers are available, with diverse payment options (cash, e-payments, credit cards, etc.)	0.40	0.09	0.21	0.34
31. Accessible restroom facilities adequately meet user needs	−0.14	0.88	0.10	0.73
30. Emergency call buttons or staff summoning devices are clearly marked and easily accessible	−0.10	0.87	0.08	0.72
29. The store provides an adequate number of rest chairs placed in convenient locations, safe and easy for seniors to use (e.g., equipped with armrests, appropriately sized)	−0.08	0.83	0.05	0.66
32. First aid kits and automated external defibrillators (AEDs) are placed in conspicuous locations	−0.04	0.81	0.04	0.65
38. Priority checkout lanes meet older adults’ needs	0.21	0.72	−0.02	0.63
35. Generous discounts for older adults	0.22	0.70	0.01	0.62
39. Delivery services meet customer needs	0.39	0.48	−0.16	0.41
2. Frequently purchased items for older adults are prominently displayed	−0.01	−0.07	0.83	0.68
3. Frequently purchased items for older adults are placed at easily accessible heights	−0.01	−0.01	0.82	0.66
1. Product categories are standardized with relatively fixed placement locations	−0.11	−0.09	0.75	0.47
11. Checkout areas provide sufficient waiting space	0.13	0.06	0.73	0.68
9. In-store floors are dry or equipped with anti-slip surfaces	0.11	0.07	0.68	0.58
10. In-store aisles are wide enough for wheelchairs or shopping carts, with no merchandise blocking pathways	0.13	0.17	0.65	0.60
8. The narrowest point of entrances/exits is wide enough for wheelchair access	0.05	0.23	0.60	0.48
4. Product variety meets daily living needs	0.12	−0.20	0.59	0.47
12. All shelves within reach for older adults to access and retrieve items	0.15	0.12	0.59	0.51
7. Entrances, exits, and in-store aisles are flat or barrier-free (e.g., gentle ramps)	0.16	0.14	0.53	0.45
Eigenvalues	12.11	4.49	1.54	
% of Variance	36.69	13.61	4.67	
% of Cumulative Variance	36.69	50.30	54.96	
Cronbach’s alpha/ McDonald’s Omega	0.921/0.921	0.898/0.898	0.904/0.904	0.939/0.932
Average of variance extracted (AVE)	0.39	0.59	0.45	
Construct Reliability (CR)	0.91	0.91	0.91	

F1: Environmental design and customer service; F2: Convenience and safety assurance; F3: Product arrangement and aisle planning.

**Table 9:** Inter factor correlation matrix of AFSI (*r*).

Factors	Factor 1	Factor 2	Factor 3
Factor 1	1		
Factor 2	0.341***	1	
Factor 3	0.756***	0.207***	1

F1: Environmental design and customer service; F2: Convenience and safety assurance; F3: Product arrangement and aisle planning. ***p < 0.001.

### 3.6 Confirmatory factor analysis

**Figure 2:**
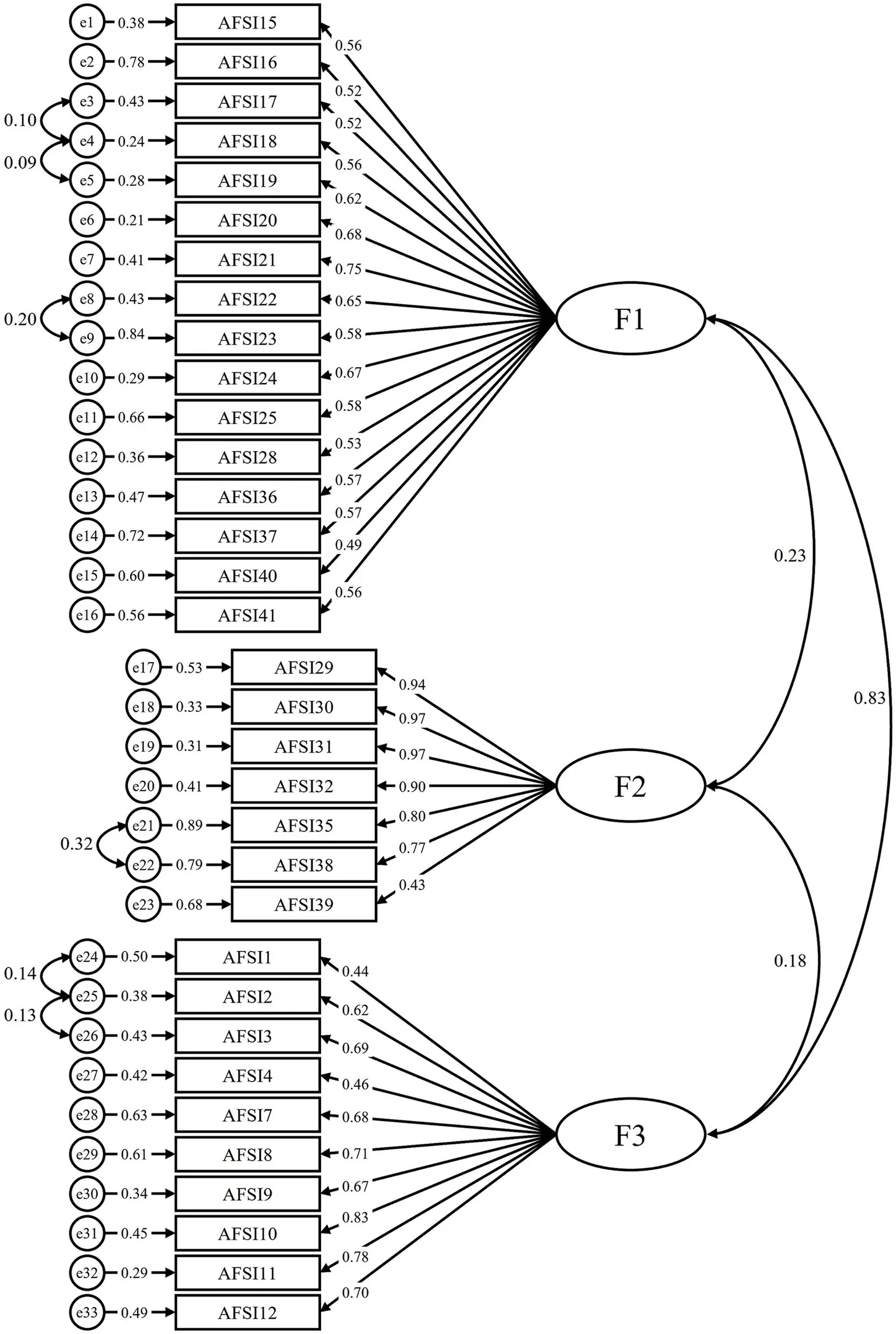
Structural equation model for the fitting model for AFSI.

CFA results demonstrated that the three-factor structure of the AFSI exhibited an acceptable model fit: $\chi_{486}^{2}$ =2.87 (*p* < 0.001), CFI = 0.879, GFI = 0.802, TLI = 0.868, RMSEA = 0.070 (90%C.I. = 0.065–0.074) and SRMR = 0.089. The factor loading of the items in CFA ranged from 0.43 to 0.97. The path diagram for the CFA model is shown in Figure 2. In MGCFA, the results showed good fit in the values of CFI, TLI, RMSEA and SRMR across comparisons by gender, age, and living status (Table 10).

**Table 10:** Multi-group confirmatory factor analysis for different sub-groups.

Model	CFI	GFI	TLI	RMSEA (90% CI)	SRMR	Model compare	ΔCFI	ΔGFI	ΔTLI	ΔRMSEA	ΔSRMR	Decision
Full sample	0.879	0.802	0.868	0.070 (0.065, 0.074)	0.089							
Gender												
M1: Configural invariance	0.869	0.777	0.858	0.042 (0.040, 0.044)	0.089							
M2: Metric invariance	0.871	0.775	0.865	0.041 (0.039, 0.043)	0.089	M1	0.002	−0.002	0.007	−0.001	0	Accept
M3: Scalar invariance	0.865	0.768	0.867	0.041 (0.039, 0.043)	0.088	M2	−0.006	−0.007	0.002	0	−0.0001	Accept
Age												
M1: Configural invariance	0.866	0.772	0.855	0.043 (0.041, 0.044)	0.089							
M2: Metric invariance	0.869	0.771	0.863	0.041 (0.040, 0.043)	0.089	M1	0.003	−0.001	0.008	−0.002	0	Accept
M3: Scalar invariance	0.869	0.767	0.871	0.040 (0.038, 0.042)	0.089	M2	0	−0.004	0.008	−0.001	0.0001	Accept
Living status												
M1: Configural invariance	0.869	0.772	0.857	0.042 (0.040, 0.044)	0.089							
M2: Metric invariance	0.871	0.770	0.865	0.041 (0.039, 0.043)	0.089	M1	0.002	−0.002	0.008	−0.001	0	Accept
M3: Scalar invariance	0.872	0.763	0.874	0.040 (0.038, 0.041)	0.089	M2	0.001	−0.007	0.009	−0.001	0	Accept

## 4 Discussion

The Age-Friendly Supermarket Index (AFSI) was developed through a systematic process comprising a literature review, two rounds of Delphi surveys, cognitive interviews, a pilot study, and a survey. The finalized AFSI consists of three domains and 33 core indicators, in addition to two supplementary indicators (see Supplementary material 1). It enables customers to comprehensively evaluate the age-friendliness of supermarkets across multiple dimensions, while also providing supermarket operators with a structured tool to assess the adequacy of their age-friendly features and identify areas for improvement.

During the Delphi survey process, experts expressed opinions regarding the measurement of certain indicators, particularly suggesting that some items, such as entrance width and shelf height, should be defined using objective standards with specific numerical thresholds. However, because respondents may have difficulty determining whether such criteria are met in practice, the AFSI was intentionally grounded in respondents’ subjective perceptions rather than objective measurements. Each indicator in the AFSI was framed as a perception-based statement and rated using a five-point Likert scale, enabling respondents to indicate the extent to which they agreed that a given feature or service met their needs. Although age-friendliness can be assessed using objective standards theoretically, perceptions of age-friendliness are inherently subjective. This approach reflects the concept of person–environment interaction and acknowledges that older adults interact with supermarket environments primarily through lived experience, and that perceived accessibility, safety, and usability may not fully correspond to technically defined standards. Moreover, this perception-based approach is consistent with existing assessment frameworks, such as the Age-Friendly City Checklist in Hong Kong (35), which relies on respondents’ subjective evaluations of each indicator. Regarding the composition of the Delphi panel, although the inclusion of an interior designer and a community organizer may appear unconventional, both provided perspectives relevant to age-friendly supermarket environments. The interior designer has experience in age-friendly home modification and universal design, enabling professional input on spatial accessibility, safety, and environmental usability for older adults. The community organizer works closely with community-dwelling older adults and was familiar with their daily shopping routines, common barriers and needs. In particular, a geriatric nurse specialist was included in the panel, and the physician worked at a community medical center serving predominantly older adults, providing clinically grounded insights into functional decline, mobility limitations, sensory changes, and age-related needs that directly influence supermarket accessibility and service design. Nevertheless, the panel lacked experts in gerontology or geriatric medicine, consumer behavior and marketing, and retail operations, which may have affected the comprehensiveness and operational applicability of the indicators. Future studies should therefore expand panel representation to include specialists from these domains to strengthen the multidimensionality of the AFSI and ensure that both aging-related considerations and retail-specific operational realities are fully incorporated.

The pilot test sample (*n* = 20) was small relative to the 41 initial items and therefore insufficient for robust item-level statistical analysis, response distribution examination, or precise timing assessment. Consequently, only preliminary internal consistency (Cronbach’s *α* = 0.881) was reported, and item performance was not evaluated quantitatively. Participant feedback was used qualitatively to refine wording and improve clarity. Future studies should conduct pilot testing with larger samples (e.g., ≥100 participants) to enable more reliable item analysis, response pattern examination, and administration time estimation before full validation.

Three factors were identified in the present study. The naming of the three factors was guided by the conceptual coherence of the items loading on each factor and their alignment with established literature. Factor 1 “environmental design and customer service” includes items related to spatial accessibility, signage clarity, and staff support, reflecting the combined influence of environmental design and customer service, focus on interior design and overall in-store comfort. Factor 2 “convenience and safety assurance” items addressing movement ease, safety features, queue management, and equipment usability. Factor 3 “product arrangement and aisle planning” comprises elements that ensure older adults can move smoothly and safely throughout the store during the shopping process. These three factors identified in the AFSI align closely with several domains of the WHO’s Age-Friendly Cities (AFC) framework (3), while also extending its application to the supermarket context. The emphasis on environmental design corresponds to the AFC domains of “Outdoor spaces and buildings” and “Community support and health services,” which highlight the importance of accessible, comfortable, and safe physical environments for older adults. The convenience and safety assurance component reflects AFC principles related to “Social participation” and “Respect and social inclusion,” as supportive services, emergency equipment, and user-friendly procedures facilitate older adults’ engagement and autonomy during shopping. Meanwhile, product arrangement and aisle planning corresponds to AFC’s focus on mobility and accessibility, ensuring that older adults can navigate retail environments with ease. However, the AFSI differs from the AFC model in several important ways. Whereas AFC guidelines are designed for broad community-level planning, the AFSI focuses on a specific micro-environment, requiring the integration of physical and social elements within operational retail contexts. The AFSI also incorporates supermarket-specific considerations, such as product placement, aisle width, equipment usability, and in-store navigation, that are not addressed in AFC guidelines but are essential for supporting functional independence during shopping. These distinctions suggest that while AFC principles provide a conceptual foundation, supermarket environments demand more context-specific operationalization. Accordingly, the AFSI contributes to extending AFC concepts into sector-specific assessment tools, offering a framework that can inform future theoretical development in age-friendly retail design.

Indicators that were not retained in the final model may be regarded as additional considerations rather than core components of age-friendliness. The eight excluded indicators (indicators 5, 6, 13, 14, 26, 27, 33, 34) were excluded for several plausible reasons. Indicators 5 and 6, which relate to the provision of designated product zones, may have limited relevance in contemporary supermarkets where such products are typically available but not organized into specific sections. Moreover, older adults often exhibit store loyalty and familiarity with product locations (36, 37), reducing the perceived necessity for dedicated zones. Indicators 13 and 14 concern stairways and escalators. Although highly important for older adults’ shopping experiences, these two indicators are not applicable to all supermarkets given that many operate on a single floor. Nevertheless, both vertical and horizontal accessibility affect the overall shopping experience. A safe, age-friendly stair and escalator designs can enhance older adults’ sense of psychological security (17). Therefore, when a supermarket has more than one floor, these two indicators should be considered essential and evaluated as independent indicators. Indicators 26 and 27 address the availability and usability of shopping baskets and carts, which are generally sufficient in most supermarkets. While carts are particularly beneficial for customers purchasing larger quantities, they are less essential for those making small, goal-oriented purchases, which may explain the item’s unclear factor loading and the resulting difficulty in assigning it to a specific domain. Finally, indicators 33 and 34 relate to the accessibility and accuracy of promotional or discount information, which is routinely displayed near products in compliance with consumer protection legislation mandating clear and accurate price information (38), rendering these features standard practice rather than distinguishing age-friendly attributes. Although these eight indicators were not included in the final AFSI, they may still serve as useful reference points in the overall design and improvement of age-friendly supermarket environments.

AFSI provides a practical implementation framework and a systematic assessment tool to guide supermarkets in the development of age-friendly environments. Several barriers may limit the adoption of a universal AFSI. In the context of Macao, the retail environment presents additional structural constraints: supermarkets typically operate within limited floor space while offering a wide variety of products, implementing age-friendly features to enhance accessibility may require reducing display or storage areas. From an operational perspective, supermarkets tend to prioritize maximizing space utilization to maintain efficiency and profitability, therefore, adoption of AFSI-related modifications that require substantial investment or reduce product display capacity may weaken willingness of supermarkets to implement changes. Furthermore, older adults’ daily activity spaces have become increasingly localized (39), and supermarket visits are primarily oriented toward purchasing staple goods (40), they prefer nearby and walkable stores. In densely populated urban areas such as Macao, where the population density reached 20,600 persons per square kilometer in 2024 (41), supermarkets with sufficient floor areas are uncommon, while those located within residential neighborhoods are unavoidably small in scale, further constraining the feasibility of implementing comprehensive age-friendly modifications.

The findings of this study also carry important policy implications for advancing age-friendly retail environments in Macao. As the AFSI provides an empirically validated and context-specific tool, it can serve as a practical reference for government departments responsible for ageing policy, consumer protection, and community development. The index may inform the development of local guidelines or accreditation schemes that encourage supermarkets to adopt age-friendly design and service standards. Given Macao’s dense urban structure and the prevalence of small-scale neighborhood supermarkets, policymakers could prioritize feasible, high-impact modifications, such as improving signage clarity, strengthening customer support services, and increase the availability of emergency assistance and first aid equipment to enhance safety, while offering incentives or technical support for more resource-intensive improvements. The AFSI also provides a framework for integrating age-friendly retail considerations into broader ageing-in-place strategies, complementing existing initiatives under the WHO Age-Friendly Cities model. By operationalizing age-friendly principles within a specific retail context, the AFSI can guide coordinated efforts between government, industry, and community organizations to promote safer, more inclusive shopping environments for older adults.

This study has several limitations. First, the AFSI does not incorporate weighted indicators. Although the current version treats all items as equally important, future research could assign differential weights based on the relative importance of specific indicators across regions, thereby more accurately reflecting older consumers’ priorities and perceptions of age-friendliness. Second, the use of convenience and snowball sampling, combine with online data collection, may have introduced selection bias by favoring older adults who are more digitally literate or have easier access to technology, despite efforts to include face-to-face interviews. The predominance of female respondents likely reflects both the gendered pattern of grocery shopping (42) and gender differences in survey participation (43, 44). Moreover, the study sample predominantly comprised older adults with relatively good IADL, with limited representation of individuals who require walking aids or who are more physically frail. Although those with restricted IADL may have had their daily necessities purchased by family members, future studies are recommended to include older adults with a wider range of mobility levels to enhance the generalizability and inclusiveness of the findings. Longitudinal designs may also help clarify how supermarkets’ adoption of AFSI-recommended improvements influence older adults’ shopping experiences, social participation, and well-being over time. Third, although RMSEA and SRMR values indicated acceptable model fit, the CFI, GFI, and TLI values were marginally below the conventionally accepted threshold (31). This pattern is common in multidimensional instruments with many items, where incremental fit indices tend to be conservative (45, 46). Absolute fit indices such as RMSEA and SRMR are considered more stable indicators of model adequacy in complex models (47). Nonetheless, future studies should consider item refinement or reduction to further improve incremental fit indices. Fourth, both EFA and CFA were conducted using the same dataset. Although independent samples or split-half validation are recommended to avoid potential inflation of model fit (48), practical constraints and the exploratory nature of this initial instrument development study necessitated the use of a single sample. Future studies should validate the AFSI using independent samples to confirm the stability of the factor structure. Fifth, although the AVE for Factor 1 and Factor 3 fell below 0.50, convergent validity remains acceptable when CR exceeds 0.60 (30). All three factors demonstrated high composite reliability (CR = 0.91), and inter factor correlations were below 0.85, supporting discriminant validity. Although PCA extracts components rather than latent factors, it was used in this preliminary stage to identify the underlying structure of the item pool. Future studies may consider re-examining the factor structure using common factor analysis methods such as principal axis factoring or maximum likelihood to further strengthen construct validity. Finally, although the results of MGCFA demonstrated measurement invariance across gender, age, and living status within the current sample, the instrument has not yet been validated in other geographic or cultural contexts. Item relevance and interpretation may vary according to regional culture, levels of social and economic development, and older adults’ expectations of supermarkets, indicating the need for further cross-regional validation studies.

## 5 Conclusion

This systematic development and validation process resulted in a comprehensive Age-Friendly Supermarket Index comprising three domains: (i) environmental design and customer service, (ii) convenience and safety assurance, and (iii) product arrangement and aisle planning. The index facilitates the identification of supermarkets or geographic areas where age-friendly development is relatively limited, thereby guiding targeted improvements. By monitoring changes in AFSI scores over time, stakeholders can assess progress and help ensure the creation and maintenance of age-friendly retail environments to better serve older customers. Nevertheless, ongoing review and adaptation of the AFSI will be necessary to reflect the specific needs, priorities, and contextual characteristics of different cities.

## Data Availability

The raw data supporting the conclusions of this article will be made available by the authors, without undue reservation.
