# Supplementary material for: The Age Friendly Supermarket Index: a Delphi study and psychometric analysis
Source: Front Public Health. 2026 Aug 28;14:1891483. doi: 10.3389/fpubh.2026.1891483 (PMC13561940; doi:10.3389/fpubh.2026.1891483)
Supplement: Supplementary file 1 [file Data_Sheet_1.DOCX]

Supplementary Material

**Age-Friendly Supermarket Index (Final Version)**

This aims to understand how you perceive the level of friendliness at **the supermarket you visited most frequently in the past month**. Please rate the supermarket based on your genuine feelings using the following criteria, with scores ranging from 1 (Strongly Disagree) to 5 (Strongly Agree).

|  | Strongly disagree | Disagree | Neutral | Agree | Strongly agree |
| --- | --- | --- | --- | --- | --- |
| **A. Environmental Design and Customer Service** | | | | | |
| 1. Shelves and walls are non-reflective | 1 | 2 | 3 | 4 | 5 |
| 2. Clear warnings are posted in wet goods areas (where seafood, meat, or vegetables are sold) or on slippery surfaces | 1 | 2 | 3 | 4 | 5 |
| 3. Store temperature is at a comfortable level | 1 | 2 | 3 | 4 | 5 |
| 4. Indoor lighting is sufficient and evenly distributed | 1 | 2 | 3 | 4 | 5 |
| 5. Air circulation is adequate and there are no unpleasant odors | 1 | 2 | 3 | 4 | 5 |
| 6. In-store signage is clear and easy to understand. | 1 | 2 | 3 | 4 | 5 |
| 7. Signage font size is sufficiently large and posted at a height suitable for older adults' vision. | 1 | 2 | 3 | 4 | 5 |
| 8. Aisle markings and directional guidance are clear, directing customers to specific areas (e.g., checkout counters, entrances/exits). | 1 | 2 | 3 | 4 | 5 |
| 9. Key areas (e.g., restrooms, service counters, entrances/exits) feature clear signage supplemented with pictograms. | 1 | 2 | 3 | 4 | 5 |
| 10. Product prices and names are clearly and legibly displayed, with signage matching the actual location of displayed items. | 1 | 2 | 3 | 4 | 5 |
| 11. Emergency exit locations are clearly marked with sufficiently bright indicator lights. | 1 | 2 | 3 | 4 | 5 |
| 12. Freezer sections are not too deep, making it easy to retrieve items | 1 | 2 | 3 | 4 | 5 |
| 13. Reasonable wait times at checkout | 1 | 2 | 3 | 4 | 5 |
| 14. Both self-checkout and staffed registers are available, with diverse payment options (cash, e-payments, credit cards, etc.) | 1 | 2 | 3 | 4 | 5 |
| 15. In-store audio announcements are clear and at an appropriate volume | 1 | 2 | 3 | 4 | 5 |
| 16. Staff are friendly and proactively offer assistance (e.g., locating products, helping with self-checkout machines) | 1 | 2 | 3 | 4 | 5 |
| **B. Convenience and Safety Assurance** | | | | | |
| 17. The store provides an adequate number of rest chairs placed in convenient locations, safe and easy for older people to use (e.g., equipped with armrests, appropriately sized) | 1 | 2 | 3 | 4 | 5 |
| 18. Emergency call buttons or staff summoning devices are clearly marked and easily accessible | 1 | 2 | 3 | 4 | 5 |
| 19. Accessible restroom facilities adequately meet user needs | 1 | 2 | 3 | 4 | 5 |
| 20. First aid kits and automated external defibrillators (AEDs) are placed in conspicuous locations | 1 | 2 | 3 | 4 | 5 |
| 21. Generous discounts for older adults | 1 | 2 | 3 | 4 | 5 |
| 22. Priority checkout lanes meet older adults' needs | 1 | 2 | 3 | 4 | 5 |
| 23. Delivery services meet customer needs | 1 | 2 | 3 | 4 | 5 |
| **C. Product Arrangement and Aisle Planning** | | | | | |
| 24. Product categories are standardized with relatively fixed placement locations | 1 | 2 | 3 | 4 | 5 |
| 25. Frequently purchased items for older adults are prominently displayed | 1 | 2 | 3 | 4 | 5 |
| 26. Frequently purchased items for older adults are placed at easily accessible heights | 1 | 2 | 3 | 4 | 5 |
| 27. Product variety meets daily living needs | 1 | 2 | 3 | 4 | 5 |
| 28. Entrances/exits, and in-store aisles are flat or barrier-free (e.g., gentle ramps) | 1 | 2 | 3 | 4 | 5 |
| 29. The narrowest point of entrances/exits is wide enough for wheelchair access | 1 | 2 | 3 | 4 | 5 |
| 30. In-store floors are dry or equipped with anti-slip surfaces | 1 | 2 | 3 | 4 | 5 |
| 31. In-store aisles are wide enough for wheelchairs or shopping carts, with no merchandise blocking pathways | 1 | 2 | 3 | 4 | 5 |
| 32. Checkout areas provide sufficient waiting space | 1 | 2 | 3 | 4 | 5 |
| 33. All shelves within reach for older adults to access and retrieve items | 1 | 2 | 3 | 4 | 5 |
| **Supplementary (Not applicable if no stairs or escalators are present)** | | | | | |
| 34. Stairs leading to the supermarket entrance or inside the store are safe (e.g., appropriate height, width, and depth; equipped with anti-slip strips or warning tape and sturdy handrails) | 1 | 2 | 3 | 4 | 5 |
| 35. The escalators leading to the supermarket entrance or inside the store are safe (e.g., equipped with emergency stop buttons, warning tape at activation points, and operating at a slower speed) | 1 | 2 | 3 | 4 | 5 |
